# Detection of Cattle Using Drones and Convolutional Neural Networks

**DOI:** 10.3390/s18072048

**Published:** 2018-06-27

**Authors:** Alberto Rivas, Pablo Chamoso, Alfonso González-Briones, Juan Manuel Corchado

**Affiliations:** 1 BISITE Digital Innovation Hub, University of Salamanca, Edificio Multiusos I+D+i, Calle Espejo 2, 37007 Salamanca, Spain; rivis@usal.es (A.R.); alfonsogb@usal.es (A.G.-B.); corchado@usal.es (J.M.C.); 2 Department of Electronics, Information and Communication, Faculty of Engineering, Osaka Institute of Technology, Osaka 535-8585, Japan; 3 Pusat Komputeran dan Informatik, Universiti Malaysia Kelantan, Karung Berkunci 36, Pengkaan Chepa, Kota Bharu 16100, Malaysia

**Keywords:** cattle detection, convolutional neural network, multirotor, drone, Unmanned Aerial Vehicle

## Abstract

Multirotor drones have been one of the most important technological advances of the last decade. Their mechanics are simple compared to other types of drones and their possibilities in flight are greater. For example, they can take-off vertically. Their capabilities have therefore brought progress to many professional activities. Moreover, advances in computing and telecommunications have also broadened the range of activities in which drones may be used. Currently, artificial intelligence and information analysis are the main areas of research in the field of computing. The case study presented in this article employed artificial intelligence techniques in the analysis of information captured by drones. More specifically, the camera installed in the drone took images which were later analyzed using Convolutional Neural Networks (CNNs) to identify the objects captured in the images. In this research, a CNN was trained to detect cattle, however the same training process could be followed to develop a CNN for the detection of any other object. This article describes the design of the platform for real-time analysis of information and its performance in the detection of cattle.

## 1. Introduction

Drones are already an inseparable part of our lives. They have important technological and functional roles in our society. Computationally and mechanically, they are equipped with electronic materials that are easy to integrate and at the same time have considerable power and precision. The simplicity of the system means that users can quickly learn how to fly multirotors and that their price is considerably low. Their high computational power allows processing thousands of commands per second, resulting in precise and controlled movements defined remotely by the user. In terms of their functionality, they are applied to a wider range of professional areas.

However, legislation must also be drawn up to regulate the new possibilities provided by technology. Many countries including the USA, the UK, Germany and Spain [1] are still drafting new guidelines and developing legislation to define the way in which multirotors can be used and the areas over which they can fly. Moreover, legislation must determine what uses are prohibited and establish licensing requirements for pilots.

This paper presents a platform that may provide solutions to some of the current legal issues. Moreover, it makes it possible to incorporate new computing and telecommunications developments to improve the performance of multirotors.

The platform is based on establishing a high-speed local network, as has already been proposed in previous works [2]. This local network is built over a set of long-range WiFi access points, which can be connected to each other if the area to be flown over is known (e.g., a city or a forest). The proposed system incorporates mechanisms that provide it with security, control of access to legally protected areas, prevention of collision with other drones connected to the platform and mechanisms that prevent the loss of network connection. In addition, all the information associated with the flight is stored and the flights can be recreated from a series of log files that are reproduced in telemetry visualization software.

Although current drones and platforms can already be utilized in many professional sectors [3,4], we have tried to provide added value to pilots by incorporating new features in the platform. The case study presented in this article used the platform and its new features to conduct a livestock census on a farm. Similarly, these developments could be used in wildlife management and for hunting.

The research described in this article presents a system for the autonomous monitoring of an area with the use of a drone (the user simply has to indicate the perimeter of the area that they want to monitor). The features newly added to the platform allow counting the livestock in the area. CNN is used to this end as it makes it possible to identify objects captured on images, and is one of the most widely used image recognition techniques in recent research [5].

A moving camera was used to acquire images in which certain elements may also be in motion (in this case, livestock). In addition to the movement of both the camera and the object of interest, several other aspects may contribute to this difficulty: the lighting conditions may vary, the background is non-static, etc. We chose to apply artificial vision techniques to deal with the changes in the numerical representation of each frame. Another problem that complicates the identification of targets may be the presence of shadows generated by animals. These situations are intractable for the majority of traditional image treatment techniques, such as Chan–Vese segmentation [6,7], which performed exceptionally well in numerous image separation tasks, but does not work with the type of data dealt with in this research.

CNNs were originally designed to solve image processing problems in which the number of inputs is variable [8,9], as in the case presented in this paper. This type of technique is based on Multilayer Artificial Neural Networks (ANNs) with a built-in feature extraction process and translational tolerance of the input image space. For this reason, CNNs are very precise at identifying targets in an image, even if the images have a lot of background noise [10]. Their precision comes from their ability to learn the distinctive features that represent the type of object that the user wants to detect, even though their relative position in the image may change.

This case study was performed over a private field, in a rural area located in the province of Salamanca (Spain) and promising results in the detection of cattle were obtained, as described further on in the article. The main novelty of the article is the application of CNNs using a drone platform that allows for the detection of cattle in flight (with 3.2 s of delay). For this reason, the drones platform and image analysis are presented separately, while their results are presented together. This novelty allows the user to obtain the results instantaneously, as they do not have to analyze the images on a remote computer. The use of the platform provides the computing capacity necessary to apply CNN while the flight is in progress.

The rest of the article is structured as follows: Section 2 reviews current state of the art in livestock detection, the use of multirotors in professional areas as well as the existing platforms. We also look into the use of CNNs in the detection and identification of targets from images. Section 3 outlines the technical aspects of the platform used in the conducted case study, this platform facilitates the application of image recognition techniques and description techniques in real-time. Section 4 describes the layout of the case study which tested the system. Section 5 presents the obtained results. Finally, Section 6 presents the conclusions from the conducted research and also discusses future lines of work.

## 2. Background

This section explores the three key areas that were the focus of this work: (i) cattle detection techniques; (ii) Ground Control Stations (GCSs) that are composed of at least one computer which runs the software required for the control and monitoring of multirotors as well as the professional areas in which multirotors can be used; and (iii) CNN, which is the main technology used in the developed platform for the detection of cattle while the flight is in progress.

### 2.1. Cattle Detection

Hunting, biological studies and farm management can all be optimized through the use of drones which allow us to detect, monitor and count different species. There have been several studies on hunting [11,12] that consider its positive and negative impacts [13] on the environment, tourism and economy. The main problem with using drones for this task is due to the existence of changing terrain (even at different seasons of the year), specie-specific features, and the distribution of the animals throughout the area under analysis [14]. In this regard, approaches based on statistical and biological methods have been published [15,16,17], which for years have tried to provide a solution to this problem, but none have had a validated commercial use. However, technological advances have made it possible to conduct studies [18,19,20] that strive to achieve the goal of automatic cattle counting.

UAVs have been used previously to monitor cattle, but the number of studies is very low. Few academic investigations on this subject have been dedicated to animal detection and counting [21], while others have focused on the monitoring of herd health [22] or feeding behavior [23], but all of them have encountered technical difficulties that have impeded their progress.

### 2.2. Professional Uses of Multirotors and Existing Ground Control Stations

Over the last five years, the use of multirotors and other type of UAVs (Unmanned Aerial Vehicles) for professional tasks has increased significantly [24]. In addition, the range of potential applications is getting wider due to the rapid advances in technology that allow reducing the size of devices and their cost. Currently, UAVs are used for social purposes, for example in search and rescue operations [25], disaster management [26] or even forest fire monitoring and measurement in an autonomous way [27]. Professional uses also appear within sectors such as surveillance [28], construction [29], agriculture [30], mapping and geology [31] or in the cinema industry [32]. This list of applications is constantly growing: insurance claim validation, wind turbine inspection, first aid, railway safety, pipeline leak detection, delivery, journalism, oil spill monitoring, and so on.

The low cost of multirotors compared to other types of UAVs used as traditional helicopters or aircraft has led to a significant increase in interest in their application. In addition to its cost, its mechanical simplicity and great functionality (such as vertical take-off and great stability) have also made the interest rise. The most common way to control them is manually via a radio-controlled transmitter that is connected directly to the multirotor; it sends signals that are processed and translated by the multirotor’s microcontroller unit into flight commands. However, the platforms that are being designed now provide the multirotor with autonomous flight modes (autopilots), meaning that their computer is the pilot and not the user.

There are three prominent platforms among the existing commercial platforms and multirotors: Mikrokopter, ArduPilot and DJI. Mikrokopter is a German company that has been manufacturing multirotors since 2006, becoming one of the pioneers in the commercial field. Initially, only manual flights were performed, but they were replaced with fully autonomous flights which are achieved through GPS and other sensors that provide stability. Further developments incorporated an onboard GPS, accelerometers, gyros and a barometric sensor for altitude control, enabling greater manoeuvrability and making autonomous flight possible. ArduPilot is one of the best known platforms, mainly because it is open source and has the largest community of collaborators on the Internet. It makes manual flights possible, but also has software (Mission Planner) that allows controlling all types of UAVs (not just multirotors) that carry one of their controllers on board (ArduPilot Mega). Since it is an open source platform, many developers have worked with it [33]. There are also studies on flying this platform indoors (e.g., [34]). DJI is a Chinese company that currently has one of the most stable multi-rotor solutions and provides numerous models and other professional filming solutions on the market.

Needless to say, there are an increasing number of platforms similar to the one presented in this article. They are used by developers from all over the world, who choose platforms depending on their needs or combine two platforms to make up for their weaknesses. In addition to dealing with different legal issues, the platform presented in this article (commercially known as Hawk Multicopter) incorporates novel functionalities. For example, it allows for multiple multirotors to be connected to one platform at the same time. To guarantee their safety, software controls the figure on the platform’s air traffic controller, avoiding collisions between drones or access to unsafe places. Similarly, the telemetry storage system is novel and can be used as electronic evidence in a trial. This article focuses on the application of artificial intelligence techniques for the detection of cattle in the images captured by an auxiliary camera, all this is done through the developed platform.

### 2.3. Convolutional Neural Networks

Deep Neural Networks are a combination of the new ANN architectures and the new learning algorithms, providing very powerful frameworks for supervised learning.

Today, CNNs are the most widely used model in image recognition because they are capable of achieving very successful results [10,35,36].

Nowadays, there are numerous convolutional neural network architectures, such as those presented in [37,38]. However, we should point to the fact that these implementations and ideas about CNNs have existed since the 1980s. In 1998, LeCun et al. [9] laid down the most important foundations of what today is known as CNNs, and focused on their application in the field of image recognition.

This type of neural network consists of three layers: (1) a convolutional layer that is responsible for applying various filters to an image to extract its main features; (2) a layer that decreases the dimensions of the image by enhancing the most important features; and (2) a layer consisting of a Multi-Layer Neural Network that is responsible for classification.

The main advantage of this type of network is that the number of parameters that must be trained is much smaller than that of a fully connected ANN with the same number of layers, thus the training is much faster.

CNNs have a wide range of applications, including the detection and erasure of faces and license plates on cars for privacy protection in Google Street View [39]. They are even able to work using the frames generated by a video for the detection of elements such as intruder detection [40].

## 3. Proposed System

This section describes the proposed system. First, we outline how any multirotor can be adapted and then connected to the developed platform and how it can benefit from all of its functionalities. Then, the software running on the GCS is detailed. The final part of this section describes the cattle detection system incorporated into the functionalities provided by the platform.

### 3.1. Connection of Existing Mutirotor Systems

One of the main features of the platform is that it is suitable for any model of the current commercial brands specialized in multirotors. Thus, any user can incorporate each of the platform features in their multirotor. For this reason, during the initial stage of system development, current systems were analyzed to enable their integration on a single platform. This was possible thanks to the design of a hardware module that is incorporated into the multirotor and makes it possible to communicate with the platform. Therefore, to connect any multirotor, it must be purchased (it costs only 30 €). The module consists of a Raspberry Pi model 3, which is a low cost and lightweight SBC (Small Board Computer), based on the Raspbian operating system (a modified version of Debian, the well-known operative system) connected to a USB WiFi antenna to connect to the deployed WiFi access points.

**Figure 1:**
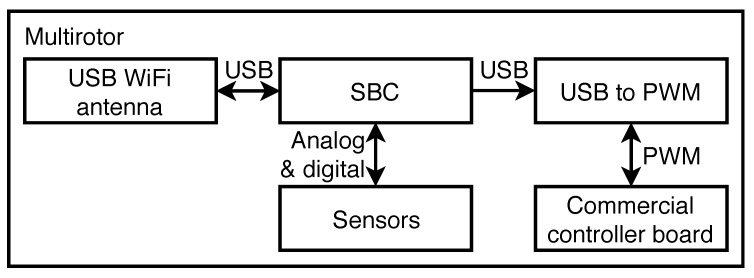
Diagram showing the connections on board.

The module is in charge of translating the digital information received from the user to PWM (Pulse Width Modulation) signals, which is the most common type of information that commercial multirotors receive to allow their control (movements, flight modes, etc.). Figure 1 shows a schema of the connections on board.

### 3.2. Ground Control Station Software

The software developed to support the work described in the article was designed to provide a solution to the different legal aspects that are still being legislated today. This is addressed by allowing for different functionalities: (i) all the exchanged information is stored in log files that can be viewed with telemetry software; (ii) a person with a controller role can view the status of all the multirotors connected to the platform to ensure that everything works correctly, similar to what air traffic controllers do; (iii) the system prevents access to restricted areas (such as airports and their vicinity) defined by the controller, which is achieved by blocking all the movements that imply access to a limited area; and (iv) it prevents collisions between the multirotors connected to the platform.

However, the description of security mechanisms is not the purpose of this article, as they have already been described in [1]. The main goal of the GCS is to offer the possibility of incorporating new functionalities in the platform, allowing pilots to take full advantage of new technological advances.

To incorporate large real-time information processing capabilities, the communication system is based on the creation of local networks based on WiFi. In this way, a large bandwidth is available to transmit all types of information in a bidirectional, encrypted and secure manner. In addition, the signal range is completely scalable and if the ground to be flown over is properly marked, a multirotor could be controlled from distances far greater than those allowed by the radio control systems currently in use.

Once a multirotor is connected to the GCS, it can be controlled with different modes: manually through a gamepad (replacing the radio stations that are traditionally used) or through the different autonomous flight modes that the GCS provides: waypoint tracking or analysis of the area within a perimeter determined by the user.

**Figure 2:**
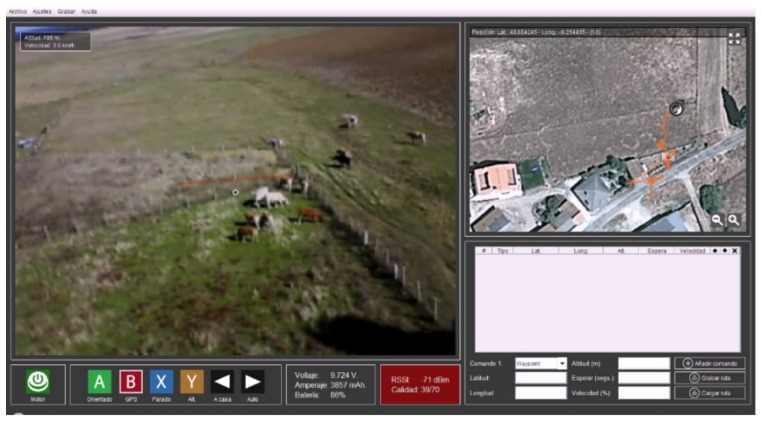
Software developed to monitor all the information in real time [41].

The developed software is responsible for managing the entire information exchange between the multirotor and the GCS, displaying the information received in real time and providing the pilot with the necessary functionality to define the behavior of the multirotor and its flight modes. An example is shown in Figure 2.

The most interesting flight mode for this case study is the autonomous flight in which the user can mark the perimeter of the area they want to travel. The multirotor will autonomously traverse this area in such a way as to ensure that it makes the optimum layout for capturing the entire area with its auxiliary camera at least once with the least possible overlap. To do this, the user must know the camera’s view frustum and introduce it into the GCS. In this case, the auxiliary camera is placed at the bottom of the multirotor, perpendicular to the ground, while the main camera is placed at the front of the multirotor.

The telemetry information is exchanged by sending and receiving messages based on the secure version of MAVLink [42,43] protocol which is transmitted using MQTT (Message Queue Telemetry Transport) [44] between a broker and two clients, one onboard the multirotor and the other included in the GCS software.

Two streaming videos are sent from the multicopter to the GCS when using both the flight view camera and an auxiliary camera to capture images perpendicular to the ground. The user can select the video that the software plays and exchange them in real time.

In addition to the software that runs on the GCS, the system has other software that allows the controller to visualize the status of the multirotors connected to the platform. It is very similar to the GCS software and allows seeing the position of each multirotor and view the parameters of the flight which are being exchanged between the GCS and the multirotor.

**Figure 3:**
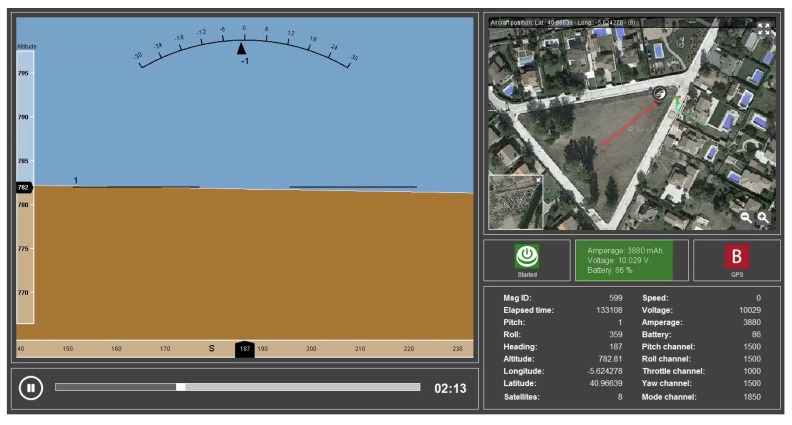
Software developed to visualize the telemetry recorded.

Software has also been developed (Figure 3) to display telemetry in the same way as for a real-time flight.

### 3.3. Cattle Recognition

To detect objects in the video taken by the auxiliary camera, individual frames are analyzed at runtime. This is done by adopting the sliding window method, which sequentially analyzes small, adjacent, and overlapping image tails positioned in a grid pattern over the frame. Every single tile is evaluated by the proposed CNN which must be trained previously. Then, the output (the possibility that there is a target in the image or background) is obtained. The dataset used to train the neural network in this case study, as well as its structure, is detailed in Section 4.

The sliding window method is reproduced at three different scales: (i) a mid-range scale, which is selected to approximate the relative size at which the targets are expected to be seen (a value which can be calculated trigonometrically based on the current flight altitude of the multirotor); (ii) then, a second scale is set at 85% of the mid-range scale window size; and, finally, (iii) the third scale is set at 115% of the mid-range scale window size. The use of more than one scale allows obtaining new information by increasing the number of total readings for every grid coordinate.

The height at which the drone flies determines the pixels that occupy a medium-sized piece of cattle in the images. The application of two windows, one larger and the other smaller, makes it possible to identify smaller and larger cattle.

The output values obtained from the CNN are processed at each of the analyzed windows. For this purpose, softmax [45] is the selected activation function, which is defined in Equation (1). $y_{i}$ represents the result of the CNN for neuron *i* of the output layer. Thus, the transformation represents the probability of any given class (represented by neuron *i*, in this case target of background). The probability value is represented by P(i).

(1) $$P\left(i\right)\equiv \sigma \left(y,i\right)=\frac{e^{y_{i}}}{\sum_{n=0}^{N}e^{y_{n}}}$$

$L_{xys}$ is also a probability value. It is defined in Equation (2), but, in this case, it represents the probability at coordinate (x;y) and scale *s* that the analyzed window belongs to the target class. When the values from the whole grid are combined for all *x*, *y* points and all *s* scales, a discrete 2D probability distribution is obtained from that corresponding input image (the frame captured by the auxiliary camera). The discrete 2D probability distribution indicates the coordinates at which target objects have been found.

(2) $$L_{xys}\equiv P\left(target|x,y,s\right)$$

(3) $$B_{xy}=\sum_{i=x-1}^{x+1}\sum_{j=y-1}^{y+1}\sum_{s=0}^{2}\left\{\begin{array}{cc} L_{ijs}, & \mathrm{if}\;L_{ijs}\geq \frac{1}{2}\\ 0, & \mathrm{if}\;L_{ijs}<\frac{1}{2} \end{array}\right.$$

Equation (3) details how the values in each coordinate are boosted. $B_{xy}$ is the boosted value. Then, a threshold (which is calculated ad hoc) is applied to the value obtained for this particular application so that a quantized representation of the probability distribution is produced. It also eliminates the majority of false positives since the ANN often finds reinforcement values in neighboring positions.

## 4. Case Study

**Figure 4:**
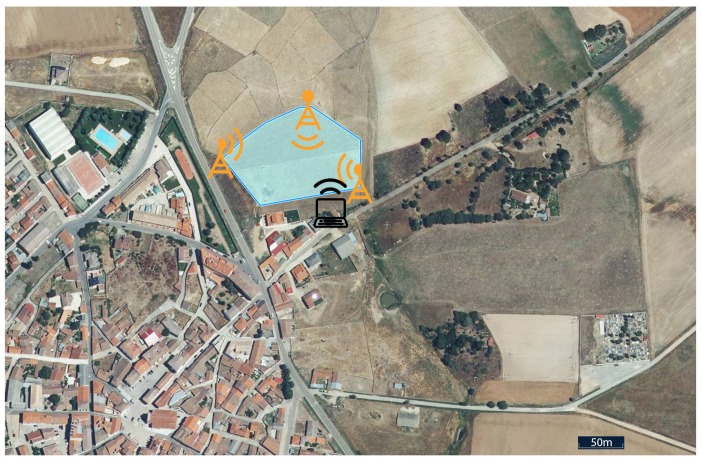
Location where the case study was conducted.

As mentioned in the introduction, the case study was conducted in the province of Salamanca (Spain) on a 1.24 ha cattle farm, in a village called La Fuente de San Esteban. The perimeter and the area of the enclosure are shown in Figure 4. To provide connectivity throughout the area, three access points were established at which directory antennas (Ubiquiti Unifi Ac Long Range 1300 Mbps) were deployed as reflected in the yellow icons in Figure 4.

The black computer icon represents the GCS, which executes the software displayed by the user. Although the platform has an offline mode, to connect to the platform and record all telemetry, Internet connection is required. In this case, a 3G router was used to send the exchanged telemetry messages and to allow the platform controller to visualize the status of the multirotor in real time. Telemetry messages, being based on MAVLink, take up few kilobytes (depending on their type), and a 30-min flight does not exceed 10 megabytes of Internet traffic (the video is not sent for privacy reasons).

The goal of multirotors is to detect animals as they search an area. Internally, they keep a record of the number of detected animals, although errors may occur in the case of groupings and the same animals crossing the path of the drone several times. However, a video with the detection display is composed and exported together with the original video. In the case of a counting error, it can easily be detected by a supervisor.

**Figure 5:**
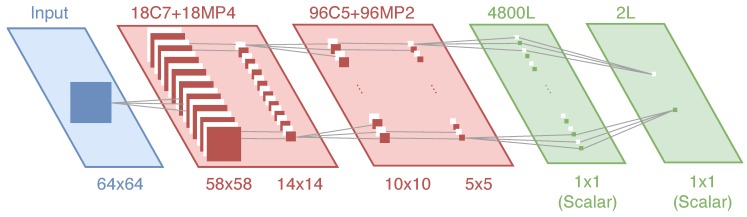
Architecture of the Convolutional Neural Network.

The architecture used by CNN for cattle detection is 64x64-18C7-MP4-96C5-MP2-4800L-2, as shown in Figure 5, wherein there is a convolutional feature extraction stage and a pooling stage, both stages are repeated two times, and finally followed by an ANN, a fully connected Multi-Layer Perceptron (MLP).

The main goal of the convolutional layer is to get the main features of the initial image. In a previously published paper [41], it was demonstrated that the architecture followed for the CNN used, provided good results. The results of the training are described again in Section 5 to facilitate the reader’s understanding. This task is done by applying a filter which is a small square matrix of input data to a complete matrix created from the original image (note that an image is a matrix of pixels with values from 0 to 255, with three channels for a RGB (Red, Green, Blue) image). This filter is slid over the original image matrix by one pixel and for every position computing an element wise operation between the two matrices. Finally, the output forms a new matrix which is called Feature Map. This process is applied to the original image with *k* filters, having as many Features Maps as *k* filters applied. *k* is the number of filters applied, in this case 18 filters for the first convolutional stage and 96 filters for the second one, as explained in Figure 5.

An additional non-linear operation is applied after the convolutional stage. The aim of this operation is to replace all the negative pixel values in the Feature Map by zero. Concretely, the operation applied is ReLU (Rectified Linear Unit [46]), which is described in Equation (4).

(4) $$f\left(x\right)=\left\{\begin{array}{cc} 0 & \mathrm{for}\;x<0\\ x, & \mathrm{for}\;x\geq 0 \end{array}\right.$$

where *x* is the value of each pixel.

Second stage is the pooling step. The goal is to reduce the dimensionality of every rectified Feature Map taking the most relevant information. This is done by applying a Max Pooling operation, which consists in defining a small window that is applied to the rectified Feature Map. Then, the largest value of the window is taken to create a new matrix that is smaller than the previous one, in this way the goal of reducing the dimensionality of the rectified Feature Map is achieved.

The last stage is a fully connected MLP ANN based on the features of convolution and pooling stages. This neural network is trained with the training dataset to finally classify the initial image among two groups, whether the images are related to cattle or not. The MLP contains one hidden layer which has 2n+1 neurons, where *n* is the number of neurons in the input layer (in this case, n=4800). The structure has been made according to the theorem of Kolmogorov [47], which states that a MLP with one hidden layer of sufficient size (2n+1) can approximate any continuous function to any desired accuracy.

**Figure 6:**
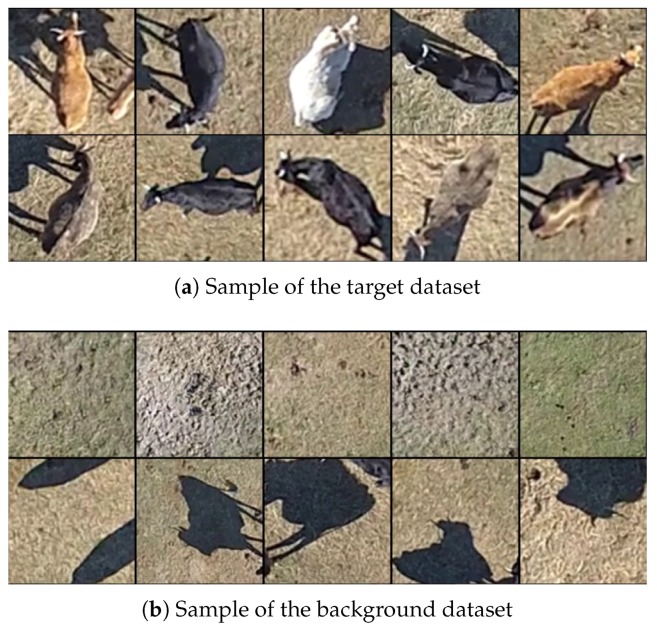
A sample of the data used to train the CNN. Two classes are used: target (**a**); and background (**b**).

As mentioned above, the training set consists of samples with the target and the background, as shown in Figure 6. Since there are no public datasets that exactly match the data we need (aerial images of livestock, their shadows and possible backgrounds), the dataset was built from recordings made by the multirotor at different heights and during different times of the year (it can influence the background color).

The animals, shadows and background have been cut by hand, totalling 100 different animals, 100 shadow images and 100 background images. Three new, 90-degree rotations have been applied to each one, creating a total of 400 images for each type and, again, slight perspective deformations have been applied to each one to generate a total of 1200 animal images, 1200 shadow images and 1200 background images.

**Figure 7:**
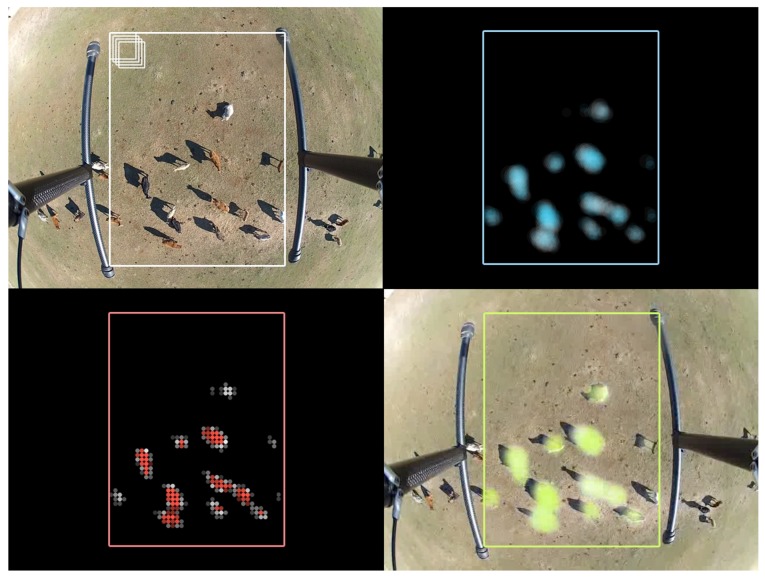
One of the frames captured by the auxiliary camera (**top-left**); analyzed by the CNN over a grid pattern producing a probability distribution $L_{xys}$ (**top-right**); values which can then be boosted and quantized as $B_{xy}$ to differentiate every single target (**bottom-left**); and visualization of the result for the user (**bottom-right**).

The different detection stages that run while the flight is in progress are shown in Figure 7. It can be seen that the result displayed by the user in the GCS highlights the detected animals.

## 5. Results

In this paper, we present a system that consists of a multirotor capable of travelling independently through an area to carry out hunting studies. The main novelty is the analysis of the images captured by the auxiliary camera of the multirotor (which is placed perpendicularly to the ground) to determine how many animals exist in the studied area. This methodology’s highly accurate results are shown below.

To evaluate the obtained results, the efficiency of the CNN must be analyzed. It should be noted that the training phase is quite fast and reaches a plateau for the training criterion (MSE (mean squared error) on classification) after just a few SGD (Stochastic Gradient Descent [48]) epochs.

13,520 images have been used, which have been divided between the images used for training (10,816 samples) and the images used for testing (2704 samples).In both Training Data Classification and Testing Data Classification there are two classes depending on the target or background. In the training process an average accuracy of 97.1% has been obtained (96.2% target sample classification and 98%) and in the testing process 95.5% (91.8% in target samples and 99.3% background samples).

In each of the analyzed frames, the number of detected animals is considered by the system. This is achieved by executing an algorithm based on connected component-labeling over the boosted and thresholded $B_{xy}$ values in the frame. The results obtained from the processing of each frame during the 3-min flight; these frames were captured by the multirotor’s auxiliary camera (while flying just above the area where most of the animals were located).

Of the total of 443 frames, collected by the camera, a total of 70 frames included cattle. Two frames per second were extracted from the video, on which the analysis was performed with three different scales, so only during 35 s the cattle were detected. Those frames were processed with the CNN which was developed in Python.

Video images were captured and broadcast with a GoPro Hero 5 in Full HD (1080 p). The image transmission itself includes a delay of 0.38 s on average (it oscillates slightly, up to ±0.153 s, with the distance the drone is at). Each of the analyzed frames occupies an average of 594 KB.

From the frames obtained in the results it becomes evident that most errors occur in overcrowded frames, when there is an average of 10 or more visible targets. In other cases, when there are fewer than 10 targets, the accuracy improves up to 98.78 percent.

Other systems that achieve great precision focus only on the detection of one target in the image, and the results obtained when comparing the most similar system with our multi-target detection proposal are also quite similar. For example, the study presented in [49], uses aerial images which are analyzed with the accuracy of 99.69 percent for the background and 98.16 percent for the targets. In [50], the accuracy varies between 77 percent and 88 percent.

**Figure 8:**
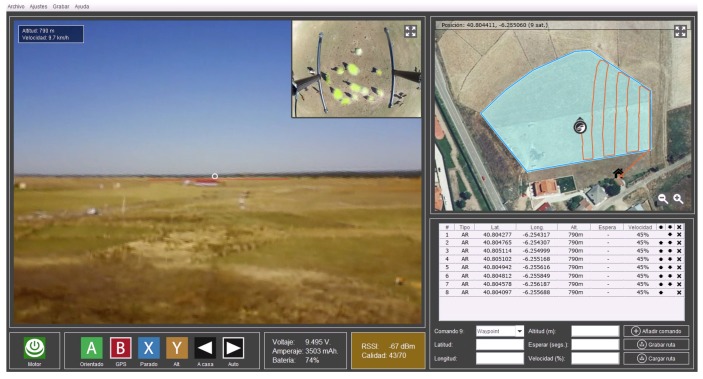
Software developed showing the analyzed video.

The image processing results are presented to the user on the GCS software, while the flight is in progress. A video is stored for subsequent viewing. Even though the results are displayed while the flight is in progress, the processing takes 3.2 s on average (with an Intel i7-8700K processor and 32 GB RAM) and there is some delay in the display of the result on the auxiliary camera (one of the reasons for which a camera is used is the delay). A screenshot and the result shown in the GCS software is shown in Figure 8.

## 6. Conclusions and Future Work

The platform allows connecting, using and monitoring multirotors of different commercial brands, and incorporates new functionalities that could for example be used in wildlife management.

The presented cattle detection system has a high success rate in the identification of livestock from the images captured by the auxiliary camera. The target recognition problem is complex because the camera is in constant movement.

A user who wishes to manage their livestock using the platform does not have to worry about controlling the flight of the multirotor, since the system guarantees that the defined area is covered in an optimal and autonomous way, adapting the height of the flight according to the characteristics of the auxiliary camera carried by the multirotor. During the flight, the system is can analyze the captured video and the one processed by the recognition system.

With regard to future work, we are currently working on a better analysis of animal groups to determine with greater precision the number of animals that are grouped together. Currently, animals are identified in clusters with an accuracy of 87 percent, but the goal is to reach an accuracy of over 99 percent. We are already working on the algorithm and intend to be publish our study once this accuracy is achieved. However, the achieved accuracy is satisfactory and slightly improves the results achieved in other studies [35], where the accuracy is of up to 85 percent.

In the same way, we will try to solve the problems that can occur when the same animal crosses several times on the path of the multirotor. The use of tracking techniques and animal identifiers (such as size and color) will be considered.

Once the problems that a multirotor may encounter in animal counting are solved, we will work on creating a distributed counting system that will be executed on several multirotors simultaneously, dividing the area to be analyzed among all participants equally.

In future work, we will not focus as much on the technical aspects, and instead will conduct an experiment in which the system will be used to detect the movement of animals and their preferred locations based on different parameters. This means that it would no longer be necessary to attach GPS devices to animals.
